# Pre-season body composition has minimal influence on in-season match availability, and match performance in female Australian Football League (AFLW) players

**DOI:** 10.3389/fspor.2022.963946

**Published:** 2022-10-26

**Authors:** Callum J. McCaskie, Marc Sim, Robert U. Newton, Jarryd Heasman, Brent Rogalski, Nicolas H. Hart

**Affiliations:** ^1^School of Medical and Health Sciences, Edith Cowan University, Joondalup, WA, Australia; ^2^West Coast Eagles Football Club, Perth, WA, Australia; ^3^School of Human Sciences, University of Western Australia, Perth, WA, Australia; ^4^Centre for Clinical Research, The University of Queensland, Brisbane, QLD, Australia; ^5^Institute for Health Research, University of Notre Dame Australia, Fremantle, WA, Australia; ^6^Faculty of Health, School of Nursing, Queensland University of Technology, Brisbane, QLD, Australia; ^7^Caring Futures Institute, College of Nursing and Health Sciences, Flinders University, Adelaide, SA, Australia

**Keywords:** anthropometric, injury, prevention, physiology, symmetry, muscle, skinfolds, testing

## Abstract

This study examined the relationship between pre-season body composition, in-season match performance, and match availability in female players competing in the Australian Football League Women's (AFLW) competition. With the outlawing of body composition assessments as part of pre-draft player evaluations in the AFLW, this study seeks to examine whether this is justified. Twenty-two (*n* = 22) players had body composition assessed with dual-energy x-ray absorptiometry at the beginning of the 2021 AFLW pre-season (whole-body and regional fat mass and lean soft-tissue mass [LSTM]). In-season match availability and match performance data (Coaches Score [CS], Champion Data Player Rank, average disposals, disposal and kicking efficiency) were collected throughout the 2021 competition. Pearson correlations were performed to assess if associations existed between body composition and in-season match performance and availability. A median split was performed to divide players into higher and lower performing groups for match performance variables. Two-sample independent *t*-tests were then used to assess differences between groups. No body composition characteristics could differentiate between in-season match availability groups (100% availability vs. <100% availability) or higher and lower performing groups for all match performance variables. Total leg LSTM asymmetry shared a moderate negative association with CS. Body composition may not be important for determining in-season match availability and performance in female AFLW players. Thus, the repercussions following the removal of pre-draft body composition assessments across the league may not be as significant as is currently perceived. Other physiological, biomechanical, or performance qualities are more variable and may mask the effect of body composition in these players. AFLW practitioners should prioritize the development of other important attributes, such as aerobic fitness, muscular strength and power, and technical skill.

## Introduction

The elite Australian Football (AF) league for females was established in 2017 (Australian Football League Women's [AFLW]) with eight teams competing. However, over the last few years, the league has rapidly expanded, and now features 18 teams. Running demands in the AFLW have been documented over the last several years (1, 2) with similar relative running intensities (distance/minute) observed with the AFL (3, 4). However, AFLW has been observed to be a tighter, more crowded game, with a greater number of tackles, errors and contested possessions observed per minute of match play compared with the AFL (5). This may help explain the differences in injury epidemiology between the two competitions with AFLW players seven times more likely to sustain an ACL injury than AFL players (6, 7) and AFLW players experiencing a greater proportion of contact injuries (8, 9). Conversely, the incidence of hamstring injuries in AFLW players are only a quarter of that of AFL players which may be due to the lower quantity of high-speed running that AFLW players undertake (2, 3). Thus, it could be suggested that the elite women's game requires different technical and physical attributes than the elite men's game.

Mitigating injury, increasing player availability, and maximizing physical performance throughout a competitive season is a key responsibility of high-performance staff. Whilst body composition characteristics have been linked with injury and physical performance in AFL players previously (10), no such association has been established with elite female AF players. Large differences in body composition characteristics have been observed between elite male and female AF players (11) which is likely due to differences in sex hormones (12), vast differences in physical match demands (4) and increased reliance on fats as an energy fuel source in females (13). Additionally, large variations in body composition characteristics have been observed for female AF players across the developmental pathway (14). Specific body composition characteristics are considered vital for physical performance as muscle mass contributes to force production, and fat mass (FM) is known to hinder thermoregulation and general locomotion (15). However, higher levels of FM may be advantageous in some sports, such as those with high in-game congestion, where contacts and collisions are a regular occurrence and total body weight is less problematic, such as the rugby codes (rugby union and rugby league).

Recently however, the AFL announced that body composition testing (*via* skinfolds) will be removed from all future pre-draft assessments across the elite men's and women's competitions (AFL and AFLW) (16). While this has been mandated by the AFL organization to reduce the psychological stigma and possible ramifications surrounding perceived body image (16), many clubs have expressed disappointment about the inaccessibility of this information until after a player has been drafted and signed despite its clear implications toward physical preparedness. Subsequently, greater insight into the importance of body composition data, particularly among female AFLW players, is crucial in understanding whether the removal of these assessments will impact future performance or match availability. Thus, the purpose of this study was to examine whether body composition characteristics were associated with on-field match performance, and in-season match availability in female players competing in the AFLW. We hypothesize that greater muscle mass and body mass will be associated with higher match availability with lower relative fat mass being associated with greater match performance.

## Materials and methods

### Study design

An observational PROSPECTIVE cohort study was used. Body composition data was obtained at the beginning of pre-season for a cohort of elite female AF players in the lead-up to the 2021 AFLW season. Pre-season training lasted for 3 months and consisted of three main training sessions per week of ~2 h in duration (resulting in ~20 kilometers (km) of total weekly running distance), two full-body resistance training sessions and individual extras (recovery, yoga, Pilates, cross-training). Match availability and match performance data were collected prospectively throughout the 2021 AFLW season. An in-season week consisted of one competitive match on the weekend, two main training sessions (~9–10 km total weekly distance) and two resistance training sessions which prioritized upper body earlier in the week and lower body at the end of the week.

### Subjects

Twenty-four elite female AF players (mean ± SD; age = 25.8 ± 4.4 years; playing experience = 3.0 ± 1.5 years; height = 169.8 ± 6.7 cm; body mass = 66.0 ± 6.7 kg) from one AFLW club participated in this study. To be eligible, players needed to be injury-free at the beginning of the competitive season (late January). This ruled two players out of the study who sustained injuries in the pre-season and missed the entire season. These players were subsequently removed from analysis. This left 22 AFLW players in the study (Table 1). Written informed consent was provided by the participating AFL club, outlining the arrangement with players to have their data collected as part of their contractual agreements for use in club operation and research endeavors. Ethics approval was provided by Edith Cowan University's Human Research Ethics Committee (ID: 2020-01055).

**Table 1:** Body composition and match performance (mean ± SD) data of all AFLW players and those within each in-season availability group.

	**All players (*****n*** = **22)**		<**100% availability (*****n*** = **12)**		**100% availability (*****n*** = **10)**	
**Descriptives**						
Age (y)	25.8 ± 4.4		26.5 ± 4.6		24.9 ± 4.2	
Height (cm)	169.8 ± 6.7		171.0 ± 6.4		168.4 ± 7.2	
Body mass (kg)	66.0 ± 6.7		65.9 ± 7.0		66.1 ± 6.8	
Playing experience (y)	3.0 ± 1.5		3.2 ± 1.7		2.8 ±1.4	
**In-season match performance and availability**						
Average coaches score	1.9 (2.5)		1.7 (3.4)		1.9 (2.0)	
Average champion data player rank	66 ± 22		62 ± 22		72 ± 22	
Average disposals	9.0 ± 3.5		7.8 ± 3.7		10.4 ± 2.9	
Kicking efficiency %	47.9 ± 12.1		48.1 ± 10.3		47.8 ± 14.4	
Disposal efficiency %	59.0 ± 9.0		60.3 ± 8.6		57.7 ± 9.7	
In-season match availability %	94.4 (22.0)		77.8 (11.0)^*^		100.0 (0.0)	
**Body composition characteristics**						
	**Absolute (kg)**	**Relative (%)**	**Absolute (kg)**	**Relative (%)**	**Absolute (kg)**	**Relative (%)**
WBLH LSTM	47.6 ± 4.4	76.2 ± 3.9	47.7 ± 5.17	76.4 ± 3.9	47.5 ± 3.56	75.9 ± 4.2
WBLH FM	13.0 ± 3.6	20.5 ± 4.2	12.7 ± 3.4	20.3 ± 4.2	13.3 ± 4.0	20.8 ± 4.4
Kicking leg LSTM	8.9 ± 0.93	71.1 ± 4.5	9.0 ± 1.1	71.8 ± 3.6	8.9 ± 0.74	70.3 ± 5.5
Kicking leg FM	3.19 ± 0.89	25.1 ± 4.8	3.05 ± 0.67	24.3 ± 4.0	3.35 ± 1.1	26.0 ± 5.7
Kicking thigh LSTM	6.36 ± 0.70	71.5 ± 4.4	6.44 ± 0.80	72.1 ± 3.5	6.26 ± 0.60	70.7 ± 5.4
Kicking thigh FM	2.4 ± 0.65	26.0 ± 4.6	2.3 ± 0.58	25.3 ± 3.8	2.4 ± 0.76	26.8 ± 5.6
Kicking shank LSTM	2.12 ± 0.28	69.7 ± 6.3	2.13 ± 0.35	70.6 ± 6.2	2.11 ± 0.18	68.6 ± 6.6
Kicking shank FM	0.74 ± 0.27	23.8 ± 6.7	0.68 ± 0.20	22.6 ± 6.6	0.80 ± 0.34	25.1 ± 6.9
**Calculated variables**						
LSTM index (kg/m^2^)	18.4 ± 1.0		18.2 ± 1.1		18.7 ± 0.9	
UB LSTM	30.2 ± 2.7	80.6 ± 4.1	30.1 ± 3.1	80.5 ± 4.6	30.3 ± 2.2	80.7 ± 3.7
UB FM	6.6 ± 2.1	17.5 ± 4.2	6.7 ± 2.2	17.6 ± 4.7	6.6 ± 2.0	17.4 ± 3.8
LB LSTM	17.8 ± 1.9	71.1 ± 4.3	17.9 ± 2.2	71.8 ± 3.6	17.6 ± 1.5	70.3 ± 5.1
LB FM	6.3 ± 1.7	25.0 ± 4.6	6.1 ± 1.3	24.3 ± 3.9	6.6 ± 2.1	25.9 ± 5.3
UB:LB LSTM	1.70 ± 0.08		1.69 ± 0.08		1.72 ± 0.07	
UB:LB FM	1.05 ± 0.16		1.08 ± 0.19		1.01 ± 0.10	
Total leg LSTM asymmetry %	2.61 ± 1.85		2.75 ± 2.35		2.43 ± 1.10	
Thigh LSTM asymmetry %	2.56 (1.83)		2.17 (2.29)		2.63 (1.50)	
Shank LSTM asymmetry %	2.67 (2.17)		2.61 (2.82)		2.72 (1.74)	

Data is presented as mean ± SD or Median (IQR) for non-normally distributed variables.

* Significantly (p < 0.002) different from 100% availability group; UB, Upper body; LB, Lower body; WBLH, whole body less head; LSTM, lean soft-tissue mass; FM, fat mass.

### Anthropometry

Height and body mass were acquired prior to undertaking the body composition assessments. Stature was recorded to the nearest 0.1 centimeter (cm) using a free-standing stadiometer (Model 217, Seca, Hamburg, Germany) with body mass measured on a calibrated weight scale (Model 22089, Seca, Hamburg, Germany).

### Body composition

Body composition was assessed using fan-beam whole-body dual-energy x-ray absorptiometry (DXA; Hologic, Horizon A, Danbury, CT, USA). Whole-body scan procedures were followed in accordance with previous work by our research team (17). Players were instructed to avoid any moderate to vigorous exercise in the 24 h prior to their scan, have emptied their bladder and arrive in a euhydrated state. All players wore their club-issued training singlet and shorts with all jewelery and metallic items removed. All whole-body data were reported with the removal of the head (WBLH; whole body less head) to maintain consistency throughout the cohort. The same qualified technician (CJM) conducted all scans and subsequent analyses. The machine was calibrated daily in accordance with manufacturer guidelines. Post-scan analysis involved the adjustment of anatomical lines to separate the various body regions including the arms, torso, and legs. The upper body (UB) was defined as everything above the iliac crest of the pelvis (excluding the head). The lower body (LB) consisted of both legs, from the feet to the femoral neck. Further sub-regions were created for the right thigh, left thigh, right shank, and left shank in accordance with our previous work (18). The coefficient of variation for the operator using the same machine in the same facility was between 0.22 and 5.09% for whole-body (total mass = 0.22%; lean soft-tissue mass (LSTM) = 0.41%; fat mass (FM) = 1.61%) and sub-regional measures (Leg LSTM = 0.95%; Leg FM = 2.36%; Thigh LSTM = 1.02%; Thigh FM = 2.27%; Shank LSTM = 1.73%; Shank FM = 5.09%). WBLH FM and LSTM were obtained along with FM and LSTM for all the sub-regions. In this study, LSTM included all fat-free soft-tissue mass and is used as a surrogate measure for muscle mass.

### Match availability

For every game in the 2021 season, the strength and conditioning specialist, physiotherapist and medical doctor collectively, would categorize each player as ‘available' or ‘unavailable'. A player was deemed available if the coaching group were able to select them, regardless of whether they played in the AFLW or the Western Australian women's state league (WAFLW) for that given week. As there are 30 contracted players on an AFLW list and only 21 players selected to play in the AFLW for a given week, some players may be required to play in the state (reserves) competition. A player was deemed unavailable if they could not be selected to play due to injury, illness, suspension, or personal reasons. No player in this study missed a game due to suspension, illness or personal reasons and were only deemed unavailable through injury.

### Match performance

#### Coaches' score

Coaches' Score (CS) are a subjective measure of match performance. The senior coach, line coaches collectively (forwards coach, midfield coach and backline coach) and the head of women's football would rate each player's performance on a scale of 0–3 (0 = poor performance and limited impact on game; 1 = played role to standard; 2 = played above expectations, good performance; 3 = exceptional performance with high impact on game). Thus, each player could receive a maximum score of 9 in a game if awarded a score of 3 by each party. Players' match performance was not rated in games in which they sustained an injury. The scale used was chosen by the club. CS have been presented as an average score received per game played.

#### Champion data player rank

Champion Data © Player Rank (CDPR) was used as an independent, and objective measure of match performance and is based on official statistics that players accumulate during a match (Champion Data ©, Melbourne, Australia). CDPR is a value based on an algorithm which considers a wide array of in-game statistics and has been developed to rate player performance and is widely accepted within the AF industry (19). The statistics that are part of the CDPR algorithm are collected in real-time by trained professionals. Slight adjustments may be required by watching a replay of the game in-depth afterwards. CDPR is weighted toward higher accumulated touches, effective use of the ball and gaining possession of the ball in a contested or disputed situation (20). CDPR are presented as an average of ranking points received per game played. CDPR was not used in games in which the player sustained an injury.

#### Average disposals, disposal efficiency, and kicking efficiency

Average disposals (AD), disposal efficiency (DE) and kicking efficiency (KE) data were retrieved from the official statistic supplier of the AFL (Champion Data ©, Melbourne, Australia). A disposal is an event whereby a player attempts to pass the ball to a teammate by kicking or handballing or is attempting a shot at goal *via* kicking (21). DE represents the proportion of disposals that each player had that were effective (the ball reached a desired teammate or went to a favorable/advantageous location). KE is a sub-group of DE and represents only kicking disposals (does not consider handballs) which also includes successful kicks at goal. Further definitions of AF statistics have been provided previously (22). Kicking and disposal accuracy/efficiency has been demonstrated to be important in the pathway to becoming an AFLW player (23). AD represents the average amount of disposals that each player had per game throughout the season. AFL coaches have been observed to perceive match performance more favorably for players with greater number of disposals and higher disposals and kicking efficiency (21). Kicking efficiency has also been previously linked with body composition characteristics in male AF players (24).

### Statistical analysis

Descriptive statistics were acquired for the cohort of players using Python (v3.7.6) in source-code editor Visual Studio code (v1.61.0). Python packages used included Numpy, Pandas, Scipy, Seaborn and Matplotlib. All variables were assessed for normality using the Shapiro-Wilk test. Variables which were not normally distributed were log-transformed before conducting further analyses. LSTM index was calculated by dividing WBLH LSTM by player height. UB to LB LSTM and FM ratios were also calculated as well as LSTM asymmetry between limbs for each LB segment (total leg, thigh, shank). Pearson correlations (r) were calculated to quantify the correlation between all body composition variables with in-season match performance and availability. The correlation matrices were created using the Seaborn package in Python. Correlation coefficients were classified as 0–0.09 = trivial; 0.1–0.29 = small; 0.3–0.49 = moderate; 0.5–0.69 = large; 0.7–0.89 = very large and 0.9–0.99 = Near perfect (25). Players were then split into two sub-groups depending on their in-season match availability [ < 100% in-season match availability (*n* = 12), and 100% availability (*n* = 10)], representing a near 50/50 split within the cohort across the two groups. Two-sample independent *t*-tests were then conducted to examine the body composition differences that existed between the two groups. Due to the large number of analyses conducted on the same dependent variables, a Bonferroni correction was applied. Subsequently, an alpha level of *p* < 0.002 was considered statistically significant for two-sample independent *t*-tests. Finally, a median-split was implemented to separate players into a higher and lower performing group according to CDPR, CS, DE, KE and AD, which is an accepted technique (26). Two-sample independent *t*-tests were conducted to assess whether body composition differences existed between the groups.

## Results

Descriptive player data for the entire group, and in-season match availability sub-groups are provided in Table 1. Significant differences between groups were observed for in-season match availability. No body composition variables significantly differentiated between in-season match availability according to sub-groups (100% season availability vs. <100% availability).

**Figure 1:**
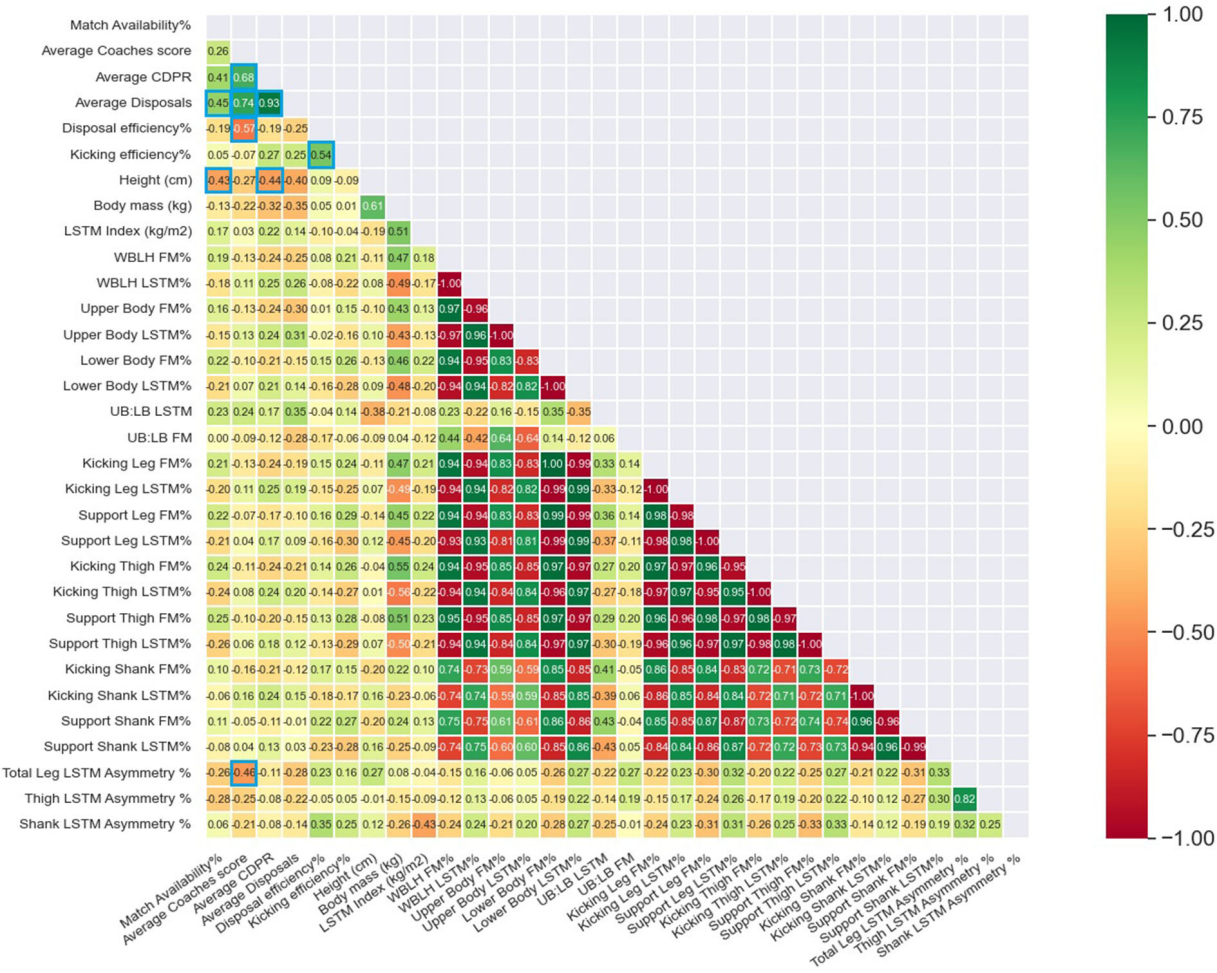
Pearson correlation matrix examining the association between pre-season body composition characteristics expressed as relative values, and in-season match availability and match performance. Significant (*p* ≤ 0.05) relationships are represented by blue boxes. CDPR, Champion Data Player Rank; LSTM, Lean soft-tissue mass; UB, Upper body; LB, Lower body; FM, fat mass.

**Figure 2:**
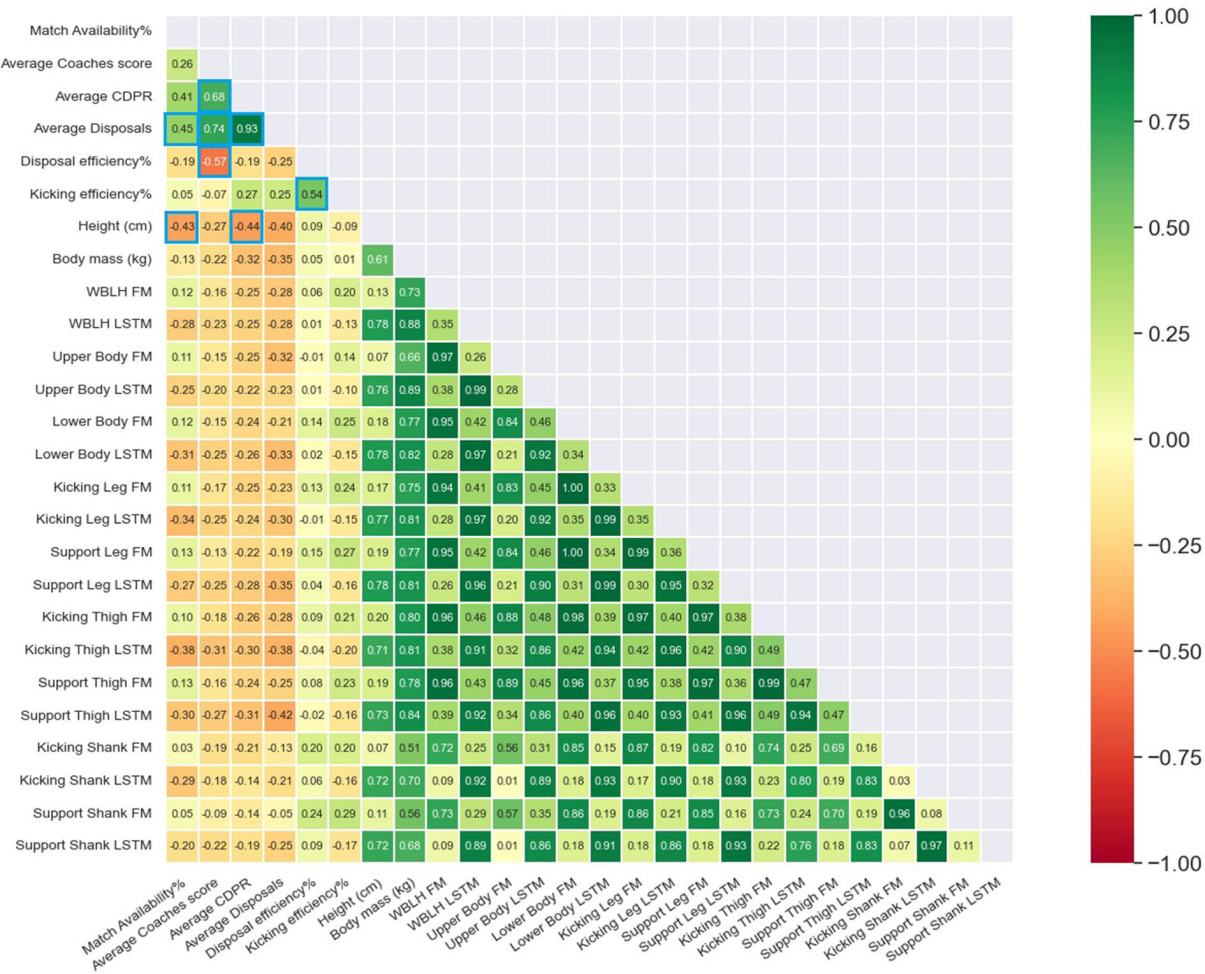
Pearson correlation matrix examining the association between pre-season body composition characteristics expressed as absolute values, and in-season match availability and match performance. Significant (p ≤ 0.05) relationships are represented by blue boxes. CDPR, Champion Data Player Rank; LSTM, Lean soft-tissue mass; UB, Upper body; LB, Lower body; FM, fat mass.

Further, no significant differences were observed for body composition characteristics between higher and lower performing groups for CDPR, CS, DE, KE or AD (Table 2). The only body composition variable significantly associated with match performance and availability was total leg LSTM asymmetry which shared a significant moderate negative association with CS (β = −0.46, 95% CI = −0.87 to−0.045) (Figure 1). In-season match performance and availability was not associated with any body composition characteristic expressed in absolute terms (Figure 2).

**Table 2:** Differences between players according to a median split for match performance variables (Champion Data Player Rank, coaches score, disposal efficiency, kicking efficiency, and average disposals).

	**<64 CDPR**	**>64 CDPR**	**<1.82 CS**	**>1.82 CS**	**<58.8% DE**	**>58.8% DE**	**<47.5% KE**	**>47.5% KE**	**<8.8 AD**	**>8.8 AD**
Age (y)	26.4 ± 4.8	25.5 ± 4.3	26.1 ± 4.8	25.4 ± 4.1	26.1 ± 3.8	25.2 ± 5.0	25.9 ± 3.47	26.1 ± 5.6	26.7 ± 5.2	24.9 ± 3.4
Height (cm)	171 ± 7	168 ± 6	172 ± 7	168 ± 6	167 ± 6	171 ± 7	168 ± 6	170 ± 8	171 ± 7	168 ± 6
Body mass (kg)	68 ± 6	63 ± 7	66 ± 6	66 ± 8	64 ± 6	67 ± 8	64 ± 6	67 ± 8	67 ± 7	65 ± 6
LSTM Index (kg/m^2^)	18.4 ± 1.1	18.4 ± 1.0	18.1 ± 0.89	18.7 ± 1.1	18.5 ± 1.1	18.3 ± 1.1	18.3 ± 0.9	18.5 ± 1.3	18.3 ± 1.1	18.5 ± 0.9
UB:LB LSTM	1.68 ± 0.06	1.73 ± 0.09	1.68 ± 0.05	1.72 ± 0.09	1.71 ± 0.09	1.70 ± 0.06	1.73 ± 0.08	1.69 ± 0.06	1.66 ± 0.04	1.74 ± 0.09
UB:LB FM	1.07 ± 0.20	1.01 ± 0.09	1.05 ± 0.18	1.05 ± 0.14	1.06 ± 0.14	1.05 ± 0.17	1.05 ± 0.16	1.03 ± 0.17	1.08 ± 0.20	1.02 ± 0.09
WBLH LSTM (kg)	48.4 ± 4.9	46.3 ± 3.7	48.0 ± 5.3	47.2 ± 3.6	46.5 ± 2.9	47.7 ± 5.4	46.6 ± 5.1	47.9 ± 3.9	48.2 ± 5.3	47.0 ± 3.5
WBLH FM (kg)	14.1 ± 2.9	11.7 ± 4.2	12.8 ± 1.7	13.1 ± 4.9	12.3 ± 4.4	13.7 ± 3.1	12.1 ± 2.6	13.5 ± 4.6	13.2 ± 3.3	12.8 ± 4.0
WBLH LSTM%	74.8 ± 3.7	77.6 ± 4.1	76.2 ± 2.6	76.1 ± 5.1	76.8 ± 4.9	75.3 ± 3.3	76.7 ± 3.8	75.8 ± 4.5	76.0 ± 3.9	76.4 ± 4.2
WBLH FM%	21.9 ± 4.0	19.1 ± 4.3	20.5 ± 2.8	20.5 ± 5.4	19.9 ± 5.2	21.5 ± 3.4	20.0 ± 4.1	20.8 ± 4.8	20.7 ± 4.2	20.3 ± 4.4
Kicking leg LSTM%	69.8 ± 3.8	72.3 ± 5.2	71.2 ± 3.0	71.0 ± 5.8	72.0 ± 5.1	70.0 ± 4.3	71.5 ± 4.3	70.7 ± 5.3	71.2 ± 3.6	71.0 ± 5.5
Kicking leg FM%	26.4 ± 4.0	23.8 ± 5.6	24.9 ± 3.2	25.2 ± 6.1	24.1 ± 5.4	26.3 ± 4.5	24.7 ± 4.6	25.4 ± 5.6	25.0 ± 3.9	25.1 ± 5.7
Support leg LSTM%	70.0 ± 3.3	72.1 ± 5.0	71.2 ± 3.0	71.0 ± 5.2	71.9 ± 4.6	70.1 ± 4.1	71.7 ± 3.9	70.6 ± 4.8	71.6 ± 3.8	70.6 ± 4.6
Support leg FM%	26.1 ± 3.5	23.9 ± 5.3	24.9 ± 3.2	25.2 ± 6.1	24.1 ± 4.9	26.2 ± 4.2	24.4 ± 4.1	25.4 ± 5.1	24.5 ± 4.1	25.4 ± 4.8
Kicking thigh LSTM%	70.0 ± 3.9	72.8 ± 4.9	71.6 ± 2.6	71.3 ± 5.9	72.2 ± 5.5	70.4 ± 3.6	72.1 ± 3.9	70.8 ± 5.4	71.5 ± 3.8	71.4 ± 5.2
Kicking thigh FM%	27.5 ± 4.0	24.6 ± 5.2	25.9 ± 2.7	26.1 ± 6.2	25.2 ± 5.7	27.2 ± 3.7	25.3 ± 4.0	26.7 ± 5.6	26.0 ± 4.0	26.0 ± 5.4
Support thigh LSTM%	70.2 ± 3.5	72.5 ± 4.8	71.4 ± 2.6	71.4 ± 5.4	72.2 ± 5.0	70.2 ± 3.5	72.0 ± 3.4	70.8 ± 5.2	71.8 ± 4.0	71.0 ± 4.5
Support thigh FM%	27.3 ± 3.6	24.7 ± 5.1	26.1 ± 2.7	25.9 ± 5.7	25.1 ± 5.3	27.3 ± 3.6	25.4 ± 3.5	26.6 ± 5.5	25.7 ± 4.2	26.3 ± 4.7
Kicking shank LSTM%	68.7 ± 6.2	70.8 ± 7.0	69.5 ± 5.9	69.9 ± 7.0	71.2 ± 5.8	68.4 ± 7.4	69.5 ± 7.3	70.2 ± 6.3	69.9 ± 5.7	69.5 ± 7.2
Kicking shank FM%	24.6 ± 6.5	22.9 ± 7.5	23.8 ± 6.1	23.7 ± 7.5	22.2 ± 6.1	25.3 ± 7.9	24.2 ± 7.6	23.1 ± 6.7	23.5 ± 5.9	24.1 ± 7.7
Support shank LSTM%	69.4 ± 5.0	71.1 ± 6.8	70.3 ± 5.3	70.5 ± 6.4	71.7 ± 5.4	69.2 ± 6.4	70.8 ± 6.5	70.1 ± 5.5	71.1 ± 5.2	69.6 ± 6.3
Support shank FM%	24.0 ± 5.1	22.5 ± 7.2	23.2 ± 5.2	23.1 ± 6.9	21.9 ± 5.7	24.5 ± 6.7	22.83 ± 6.7	23.24 ± 5.8	22.3 ± 5.22	24.0 ± 6.79
Total leg LSTM asymmetry %	2.43 ± 1.25	2.31 ± 1.86	2.99 ± 1.96	2.22 ± 1.74	2.13 ± 1.81	2.79 ± 1.15	1.91 ± 1.40	2.98 ± 1.54	3.40 ± 2.18	1.81 ± 1.03
Thigh LSTM asymmetry %	2.51 ± 1.18	2.82 ± 2.49	2.95 ± 2.58	3.00 ± 2.20	3.01 ± 2.32	2.57 ± 1.24	2.49 ± 1.10	3.00 ± 2.47	3.86 ± 2.92	2.08 ± 1.09
Shank LSTM asymmetry %	3.02 ± 2.25	2.92 ± 2.43	3.01 ± 2.09	2.90 ± 2.44	2.84 ± 2.57	3.27 ± 2.12	2.70 ± 1.25	3.43 ± 3.04	3.40 ± 2.90	2.51 ± 1.24

CDPR, Champion Data ^®^ Player Rank; CS, Coaches Score; DE, Disposal efficiency; KE, Kicking efficiency; AD, Average disposals; UB, Upper body; LB, Lower body; WBLH, whole body less head; LSTM, lean soft-tissue mass; FM, fat mass.

## Discussion

This study investigated whether start of pre-season body composition characteristics were associated with in-season match performance, and match availability in elite female AF players. No body composition characteristics differentiated between the availability of athletes or between higher and lower performing players for CDPR, CS, DE, KE and AD. The AFLW season consisted of nine games across nine consecutive weeks from 29th January−28th March (not including finals). In comparison to the male league (AFL; 22 games), and other elite women's competitions, such as the Football Association women's Super League (Soccer; 22 games), the AFLW season is markedly shorter. Thus, a short season may reduce the relative influence of body composition as a notable contributing injury risk factor and match performance indicator in AFLW players. Further, due to the competition's recent establishment, the average playing experience across the cohort was only 3 years, which may highlight the limited capacity to detect a relationship here. Recent research discovered elite female senior AF players had superior intermittent running performance, sprint speed, vertical jump height and greater performance on technical kicking and handballing skill tests than their non-elite counterparts (14, 23). Thus, other factors such as muscular strength, aerobic fitness, technical skill, and pre-season training load exposure may be more important when examining in-season match performance and availability in AFLW players (14, 19, 27). While it has been suggested that FM has a negative effect on general body movement (28) and kicking accuracy in AF (24), the influence on match performance and availability across a nine-week season in women AF players (AFLW) appears minimal.

Greater body mass has previously been linked with lower injury risk in an elite male AF cohort, with every additional 1 kg of body mass decreasing injury risk by 11.3% (29). Whilst elite AF players cover ~13 kilometers (km) per game (3), players are also exposed to frequent heavy collisions which also places them at risk of contact injuries (10). It is likely that sufficient body mass (comprising muscle and fat) is required to absorb these forces and protect players from injury. Given the elite women's game is characterized by a greater proportion of contact injuries (9), and more contested possessions, tackles and stoppages per minute of play than the elite men's game (all of which increase the frequency of collisions) (5), it was hypothesized that greater muscle and body mass (as opposed to its composition) would be associated with higher match availability and performance (5, 30). However, in our study, no body composition characteristic, including total body mass, was associated with in-season match availability, suggesting other factors may be more influential in this relationship.

Similarly, no significant differences in body composition characteristics were observed between those players who were available for the entire season (100% availability) and those who missed at least one game due to injury throughout the season (<100% availability). This is in agreement with a study in elite professional rugby which found no relationship between body composition and injury (31). Conversely, whole body FM% has been positively associated with injury in male soccer players (32, 33). As the AFLW is a new and emerging competition with a current lack of developmental pathways, the league features many players who previously played other sports (including netball, basketball, and Gaelic Football). Thus, highlighting the fact that many players have not had the exposure to longitudinal AF specific loading and training history, which may place them at a greater risk of injury. Factoring this into the analysis may have provided more insight into this relationship. Further, females in the AFLW typically cover 50–70% less high-speed running distance per minute of match play than their elite male AFL counterparts (2, 3). As high-speed running induces neuromuscular fatigue and is considered a common mechanism for hamstring injury (34), AFLW players may not be at the same risk as elite male players comparatively. While body composition assessment has been banned from AFL and AFLW pre-draft evaluations for other reasons, the results of this study indicate that body composition evaluated at the beginning of the pre-season for AFLW players may not be as important to match performance and in-season availability as may currently be perceived. This potentially highlights the greater importance of other attributes including aerobic fitness, and muscular power and strength with injury. Nonetheless, these findings present important insights for AFLW practitioners.

In the current study, match performance was determined both subjectively by the coaches and football department based on their perception of each player's impact on the game (CS) and objectively *via* CDPR, AD, DE and KE. However, no body composition characteristic could differentiate between higher and lower performing players for any match performance metric. As FM is known to impair cardiorespiratory performance by acting as “dead weight” and not contributing to force production and movement (28), it was hypothesized that lower levels of FM may allow players to cover more ground during a game, increasing their likelihood of having a greater impact on the game. By the same token, it was further conjectured that lower levels of FM may delay the onset of fatigue, lowering the risk of injury during a match. However, our data does not support such hypotheses. One explanation could be that the influence of FM in AFLW players is reduced due to the shorter match duration (~80 mins of match play vs. 120 mins in the AFL) and lower running volume [~6 vs. ~13 km (1, 3)] during competitive match play. Further, strength, power and technical skill are not as developed in the women's game and this is likely due to the disparities in training opportunities, development pathways, financial support and access to staff and facilities (35). Thus, it is likely that variations in these factors may overwhelm any minor influences that body composition has on in-season match performance and availability. This provides important insights for practitioners who should look to prioritize the development of other important attributes over specific body composition traits.

Interestingly, total leg LSTM asymmetry was the only body composition characteristic associated with any match performance metric (which shared a moderate negative relationship with Coaches Score). Research examining the relationship between LSTM asymmetry and sporting performance outcomes is scarce. Hart and colleagues (36) demonstrated that sub-elite AF players with greater kicking accuracy had significantly less leg LSTM asymmetry. However, LSTM asymmetry was not associated with kicking or disposal efficiency in the current study. LSTM asymmetries have been shown to influence jumping performance in collegiate athletes previously (37), but how this translates to match performance outcomes is unclear.

To our knowledge, this is the first study which has examined the relationship between pre-season body composition characteristics with in-season AFLW match performance and match availability. However, several limitations of this research are worth noting. First, body composition assessments were undertaken at the beginning of pre-season, roughly 3 months prior to the beginning of the competitive season. Thus, this may not be a true reflection of players' kinanthropometric profile throughout the competitive season as notable changes in body composition are likely to occur through targeted intervention across pre-season. Thus, start of pre-season body composition may be more a reflection of players' compliance to their off-season fitness program. Additionally, our results are delimited to 22 players at this one point in time, involving factors across one season. Thus, reducing the statistical power of the analyses. Smaller squad numbers (in comparison to elite male teams), contractual arrangements (which limits their time at the club), and the COVID-19 pandemic made data collection challenging. Future research should be directed to researching players over multiple teams and multiple seasons and multiple time points in the year while also considering the positional requirements and training history of each player. The results of this study also may not necessarily apply to other AFLW teams as all teams have varying levels of experience and an array of players who have crossed over from a variety of other sports.

Ultimately, no start of pre-season body composition characteristics were associated with in-season match availability, or most match performance metrics in female AF players playing in the AFLW. As the AFLW has only recently been established, other factors such as technical skill level, neuromuscular and cardiorespiratory capacities, and training history may share a greater association with in-season performance and availability. Body composition assessments have been banned as part of pre-draft evaluation of potential recruits and this study may provide justification that body composition is not as important as other physical and technical attributes in AFLW players. As the AFLW competition is still in its infancy, it's likely the physical and technical attributes are more variable between players and mask any influence that body composition has on in-season match availability and performance. As such, further research is needed to uncover the specific attributes linked with in-season match availability and performance in female AF players.

## Data availability statement

The datasets presented in this article are not readily available because due to the agreement with the football club, no data outside of what has been illustrated in the manuscript can be made publicly available. Requests to access the datasets should be directed to c.mccaskie@ecu.edu.au.

## Ethics statement

The studies involving human participants were reviewed and approved by Edith Cowan University Human Research Ethics Committee. The patients/participants provided their written informed consent to participate in this study.

## Author contributions

CM, NH, MS, RN, JH, and BR conceived and designed the research. CM, JH, and BR collected the data. CM analyzed the data with assistance from NH, MS, and RN. CM wrote the manuscript with assistance from all authors. All authors read and approved the manuscript.

## Conflict of interest

Authors CM, JH, and BR were employed by West Coast Eagles Football Club. The remaining authors declare that the research was conducted in the absence of any commercial or financial relationships that could be construed as a potential conflict of interest.

## Publisher's note

All claims expressed in this article are solely those of the authors and do not necessarily represent those of their affiliated organizations, or those of the publisher, the editors and the reviewers. Any product that may be evaluated in this article, or claim that may be made by its manufacturer, is not guaranteed or endorsed by the publisher.
